# Supplementary figures and images for: Effects of a natural precipitation gradient on fish and macroinvertebrate assemblages in coastal streams
Source: PeerJ. 2021 Oct 1;9:e12137. doi: 10.7717/peerj.12137 (PMC8489409; doi:10.7717/peerj.12137)

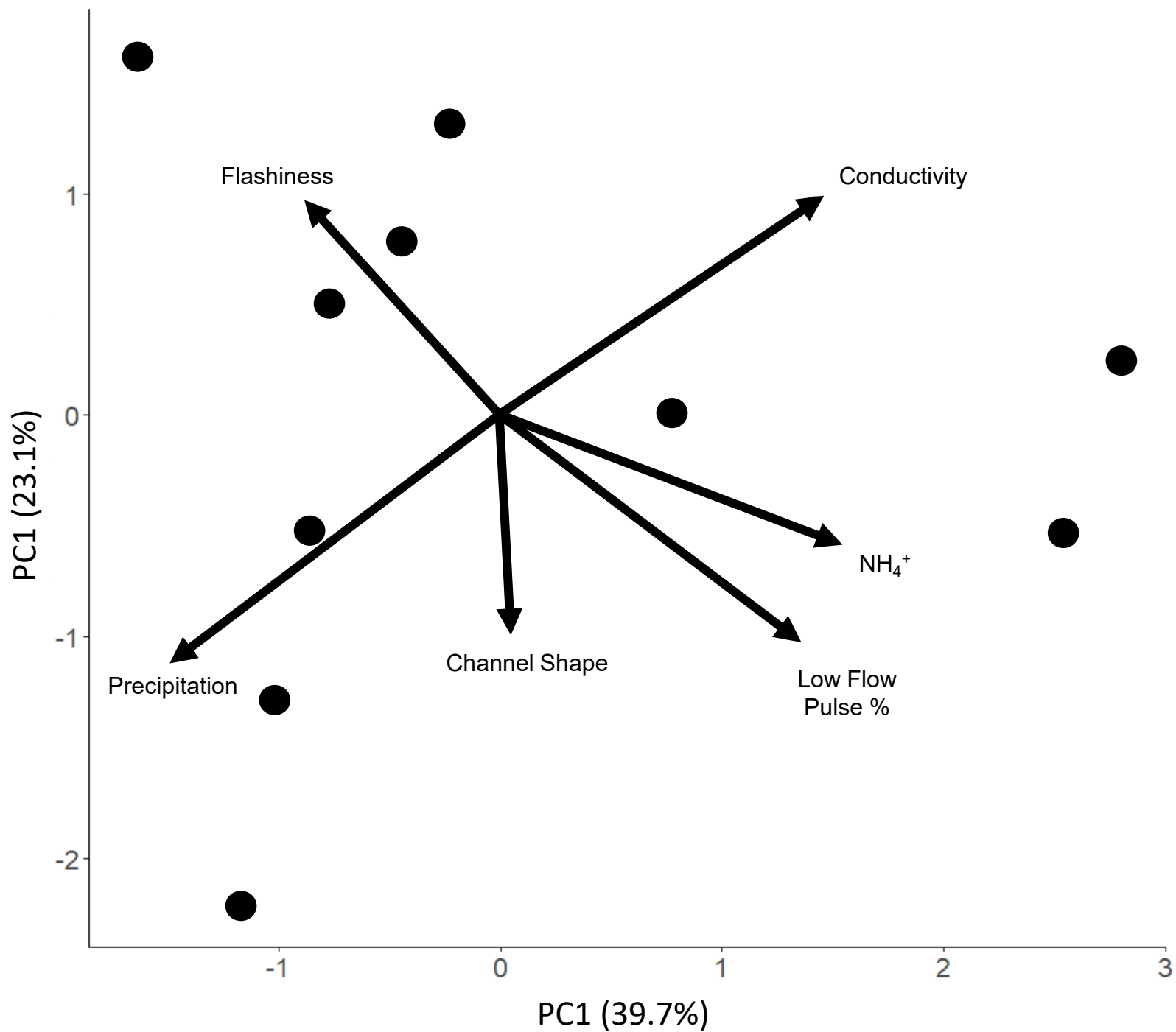

Supplement: Supplemental Information 1 — Circles representing sample sites are colored based on their annual precipitation. Axes labels include the percentage of variance explained by the first principal component (horizontal axis) and the second principal component (vertical axis). [file peerj-09-12137-s001.pdf]

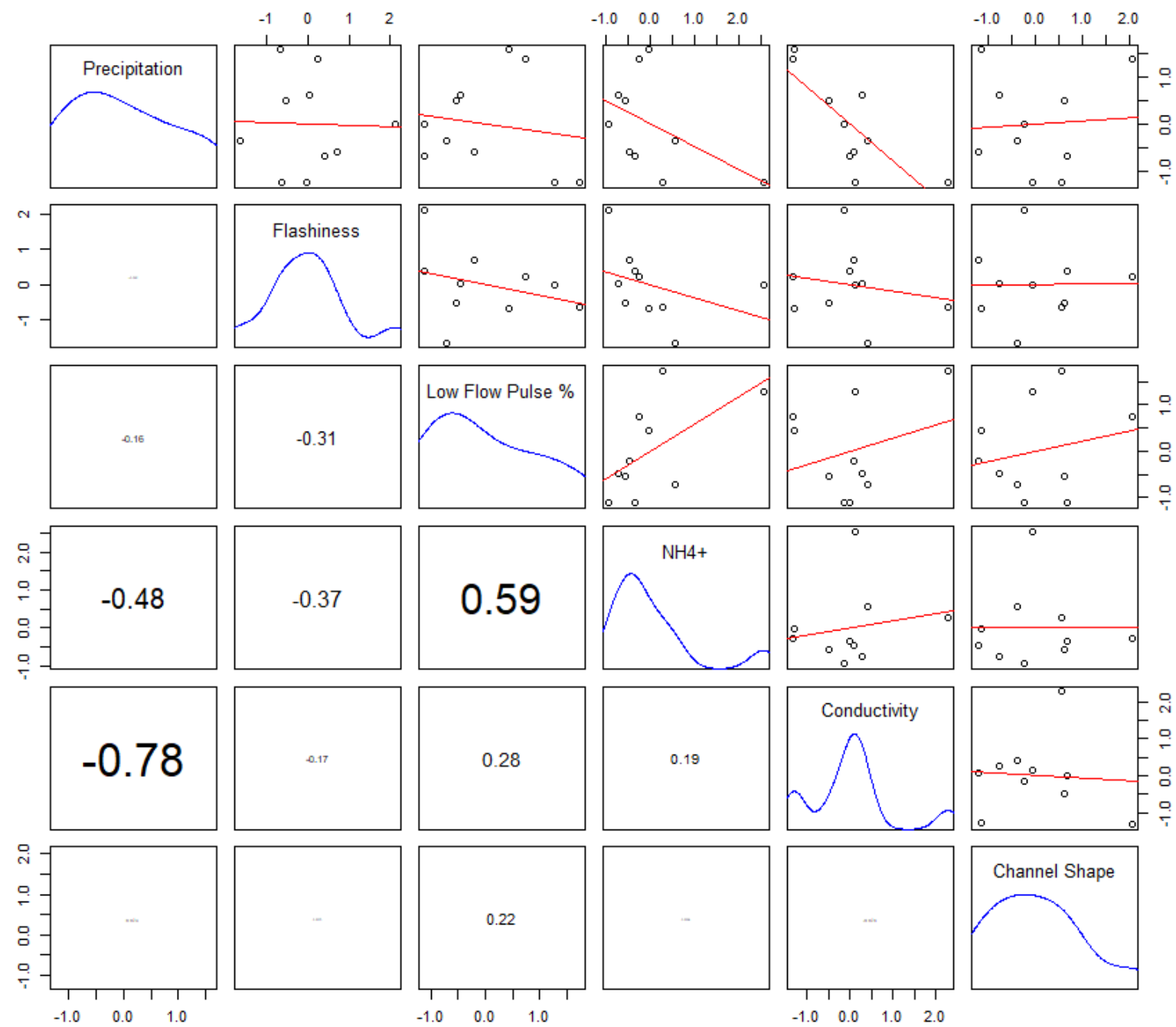

Supplement: Supplemental Information 2 — Scatter plots with linear regression are depicted in the upper right panels. Data point density is plotted by the blue line along the center diagonal panels. Correlation coefficients are scaled by magnitude and depicted in the lower left panels opposite of their complimentary scatterplot in the top right section. [file peerj-09-12137-s002.pdf]

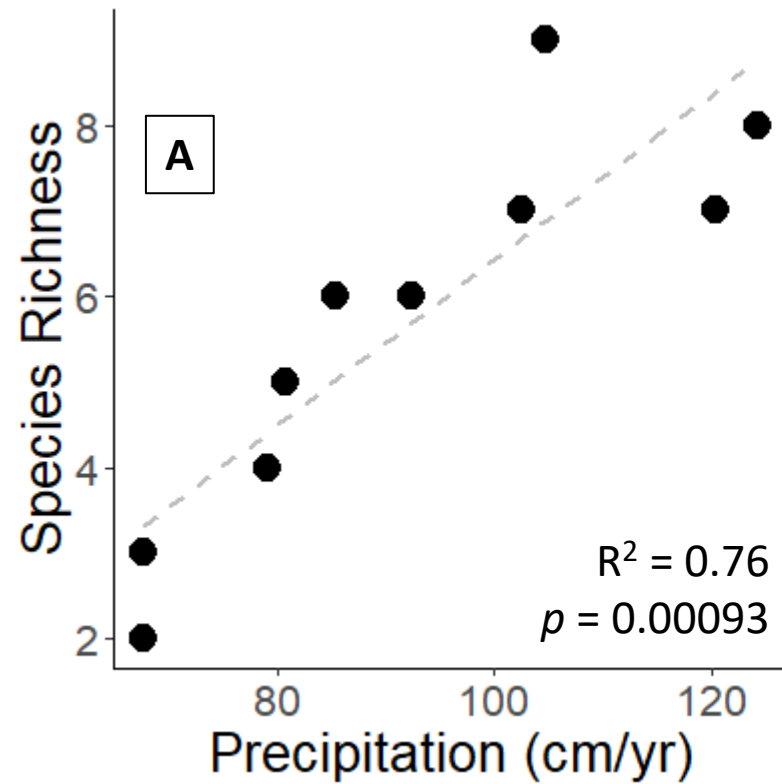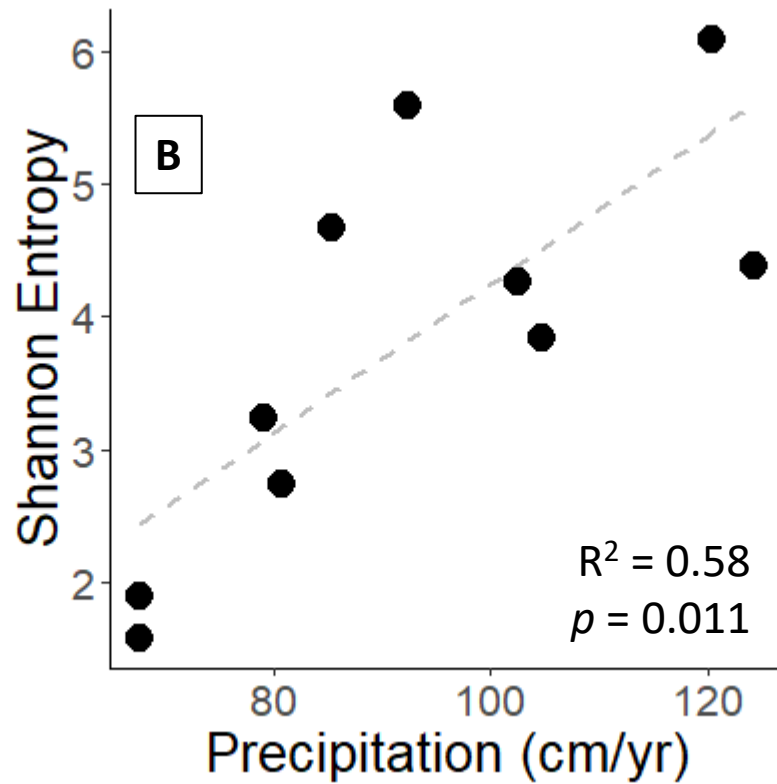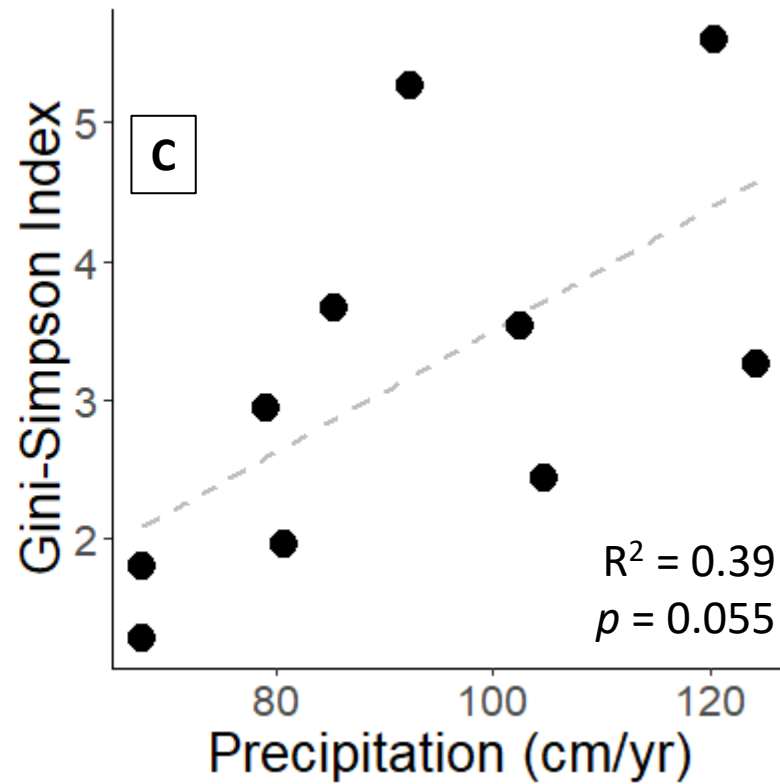

Supplement: Supplemental Information 3 — Dotted grey lines depict linear regressions and the associated correlation coefficients and p-values are printed in the lower right section of each panel. [file peerj-09-12137-s003.pdf]

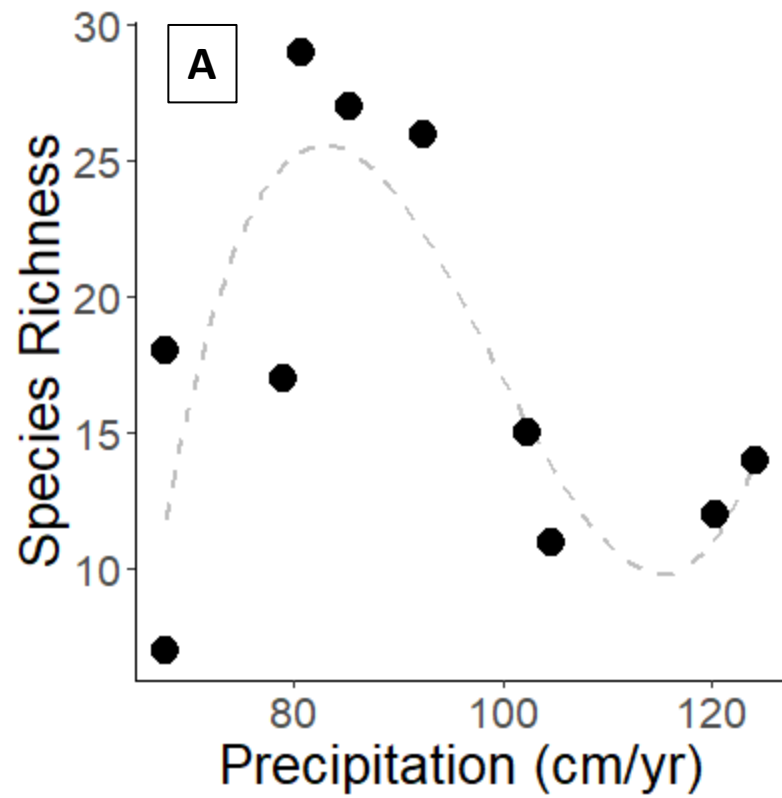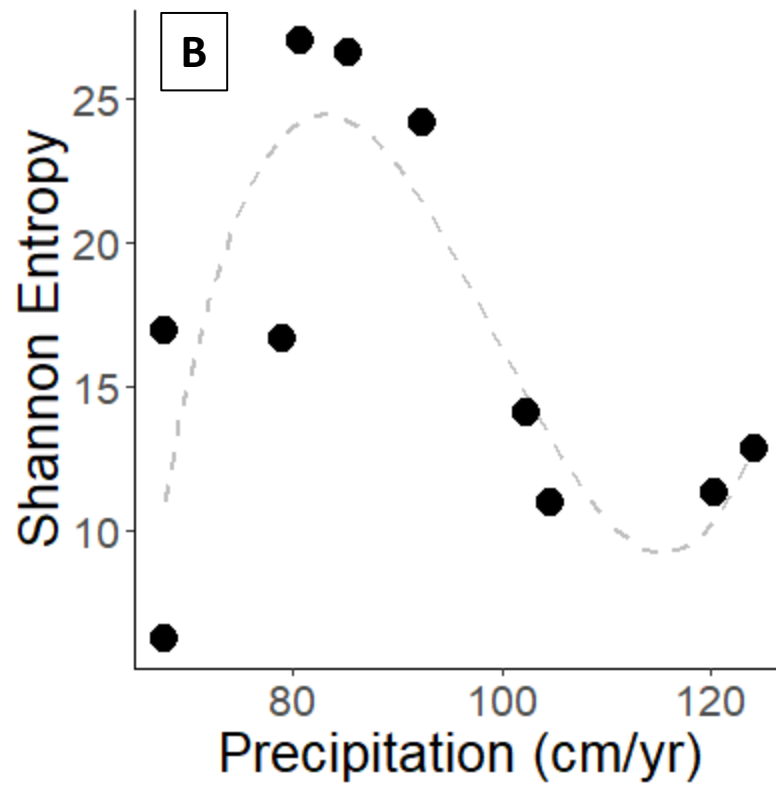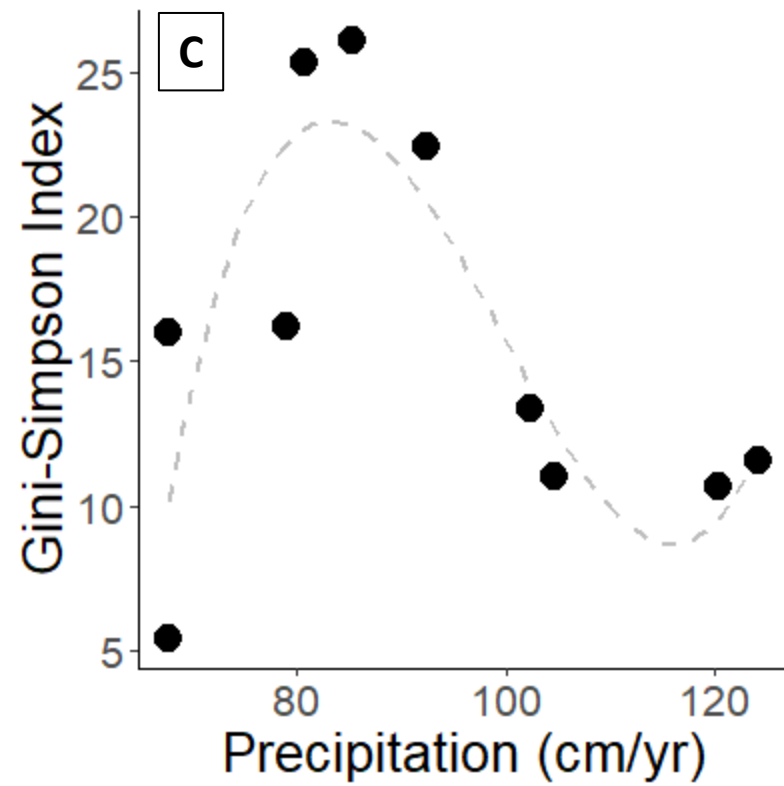

Supplement: Supplemental Information 4 — Dotted lines represent a fitted parabolic regression to aid in visualization of the trend along the gradient. Invertebrate biodiversity does not exhibit a consistent relationship with annual precipitation, but the highest invertebrate diversity was observed at sites within the middle of the rainfall gradient. [file peerj-09-12137-s004.pdf]
